# French Versions of 4 English Questionnaires on Problematic Smartphone Use: Cross-Cultural Linguistic Translation and Adaptation Study

**DOI:** 10.2196/53958

**Published:** 2025-02-26

**Authors:** Islam El Boudi, Mathilde Riant, Alexandre Bellier, Nicolas Vuillerme

**Affiliations:** 1 Faculty of Medicine AGEIS, Grenoble Alpes University Grenoble France; 2 Inserm CIC 1406 Grenoble Alpes University Hospital (CHUGA), Grenoble Alpes University 38000 Grenoble France; 3 Institut Universitaire de France 75005 Paris France

**Keywords:** problematic use, smartphone, French questionnaire, linguistic translation, forward/backward process, mobile phone

## Abstract

**Background:**

Excessive use of smartphones is recognized as a major problem in our modern society and can have dramatic consequences on the health of adolescents and young adults. Measuring problematic smartphone use in research and clinical practice is generally operationalized with self-reported questionnaires. In order to comprehensively assess the issue of problematic smartphone usage within the French population, it is imperative to employ validated French-language questionnaires. However, at this point, existing questionnaires are primarily available in English. Furthermore, to the best of our knowledge, these English questionnaires have yet to undergo validation processes for French-speaking cohorts.

**Objective:**

The aim of this study was to perform a cross-cultural translation of the Smartphone Addiction Scale, Nomophobia Questionnaire, Problematic Use of Mobile Phones scale, and Smartphone Addiction Proneness Scale to French.

**Methods:**

The translation process was performed using the forward/backward method. The first translation phase involved asking 4 independent French translators to translate the original English version of the questionnaires into French. In the second phase, the French version was backtranslated to English by a native English speaker. In the third phase, 2 concept experts were asked to comment and suggest modifications to the statements if necessary. Finally, the last version of the translated questionnaires was presented to 18 participants to assess the clarity, intelligibility, and acceptability of the translations.

**Results:**

During the forward translation step, the translation differences were minor. During the backward translation, the English native speaker correctly backtranslated 18 of the 33 items of the Smartphone Addiction Scale, 17 of the 20 items of the Problematic Use of Mobile Phones scale, and 13 of the 15 items of the Smartphone Addiction Proneness Scale. Backtranslation for the Nomophobia Questionnaire was less satisfactory, with only 10 out of 20 items that were correctly backtranslated. The linguistic verification step revealed a minimal modification for the 4 questionnaires. The participants also suggested few improvements that we have considered for the final version. We produced the final version directly after this step.

**Conclusions:**

We successfully adapted and effectively translated 4 questionnaires that assess problematic smartphone use to French. This step is a prerequisite for the validation of the French questionnaires. These adapted measures can serve as valuable research instruments for investigating and addressing issues related to problematic smartphone use in French-speaking countries and for making international comparisons.

## Introduction

Today, the excessive use of smartphones and screens, in general, is recognized as a major problem in our society. Recent world events that have led to individuals being confined to their homes for several months have intensified the amount of scientific and political concerns surrounding this issue. Excessive smartphone use can have dramatic consequences for the health and cognitive development of adolescents and young adults [1]. Indeed, difficulty in regulating smartphone use can lead to serious pathological disorders such as addiction to video games [2].

Although health care professionals, researchers, and politicians recognize that excessive smartphone use is problematic, its legitimacy as a pathological disorder is not yet recognized by the American Psychiatric Association [2]. For this reason, the notion of problematic smartphone use is favored by a large number of researchers. Problematic smartphone use does, however, refer to symptoms of dependence such as loss of control, overuse, increased tolerance (ie, spending more time on the smartphone to be satiated), or withdrawal symptoms once individuals are no longer in possession of their smartphones [3].

In order to identify individuals at risk of problematic use, the measurement of smartphone use practices is generally operationalized in research by using self-reported questionnaires. Three very recently published systematic reviews [3-5] identified 4 measurement scales most commonly used to measure problematic smartphone use, 3 of which are used with young adult students, that is, the Smartphone Addiction Scale [6], the Nomophobia Questionnaire [7], and the Problematic Use of Mobile Phones scale [8], and 1 for adolescents, that is, the Smartphone Addiction Proneness Scale [9]. What these 4 scales have in common is that they assess problematic smartphone use across several dimensions, often inspired by the fourth and fifth versions of the Diagnostic and Statistical Manual of Mental Disorders (DSM-IV and DSM-V) [2,10].

The Smartphone Addiction Scale was designed by Kwon et al [6] based on the diagnostic criteria for internet gaming addiction in DSM-IV [10]. It measures problematic smartphone use along 6 dimensions: disruption of daily life due to smartphone use, positive anticipation of use, withdrawal symptoms, overuse, tolerance, and importance of the virtual world for social relationships. This scale has the advantage of being validated in several languages, including English [6], Arabic [11], Brazilian [12], Malaysian [13], and Persian [14]. It also has good cross-cultural consistency, with Cronbach α ranging from 0.93 to 0.97. Although the Smartphone Addiction Scale has been translated and validated in French in a shorter 10-statement version [15], the original 33-items scale has not been translated and validated in French.

The Nomophobia Questionnaire was designed by Yildirim and Correia [7] and measures individuals’ fear of being separated from their smartphones. It measures 4 dimensions of smartphone separation fear: not being able to communicate, losing connection with the outside world, not being able to access information, and giving up comfort. According to the research work of Jahrami et al [16], this questionnaire has been translated into 7 languages (eg, Chinese [17], Italian [18]) and consistently has very good internal consistency (0.88≤α≤0.96).

The Problematic Use of Mobile Phones scale, designed by Merlo et al [8], draws heavily on the criteria for online gaming addiction in DSM versions IV [10] and V [2]. This scale considers 10 dimensions of problematic smartphone use in young adults: tolerance, withdrawal symptoms, smartphone use longer than expected, time spent on the smartphone, irrepressible desire to use the smartphone, abandonment or reduction of other activities, smartphone use despite physical and psychological consequences, smartphone use in risky situations, smartphone use despite social consequences, and inability to fulfill obligations due to use. Although being less validated, this scale has been translated into English [8], German [19], and Arabic [20], and its consistency appears very satisfactory (0.90≤α≤0.94).

To date, 2 other scales measuring problematic smartphone use by young adults have been validated in French. The first scale, that is, the Implicit Association Test (smartphone) [21] is strongly inspired by the internet addiction test developed by Young [22] and not according to a set of diagnostic criteria for problematic smartphone use. However, the factor structures and internal consistency of the scale (α=0.93) were good. The other scale, that is, the Problematic Mobile Phone Use Questionnaire by Lopez-Fernandez et al [23] is divided into 3 categories and has good internal consistency: dangerous use (α=0.81), prohibited use (α=0.74), and dependent use (α=0.90). The strength of this scale lies in its cross-cultural validity (eg, French, German, Hungarian, English, Finnish, Italian, Polish, Spanish), but surprisingly, it is one of the few scales that is not based on any version of DSM. However, recent research has shown that taking inspiration from gaming disorder criteria to measure problematic smartphone use is a way to better understand this phenomenon [24].

The Smartphone Addiction Proneness Scale by Kim et al [9] is inspired by the assessment criteria of Young’s [22] Internet Addiction Test scale. To our knowledge, it is one of the few scales designed to measure problematic smartphone use in adolescents. The Smartphone Addiction Proneness Scale assesses 4 main dimensions: craving symptoms, tolerance, predominance of virtual life, and disruption of coping in daily life. To date, it has been translated into Korean (ie, translated from English) [9], Malaysian [25], German [26], and Chinese [27]. The interrater internal consistency of this Korean scale is consistently acceptable (α=0.88).

Given the importance of completing proposals for measuring problematic smartphone use based primarily on the findings of DSM and to better understand this societal issue, the aim of this study is to linguistically translate the Smartphone Addiction Scale, the Nomophobia Questionnaire, the Problematic Use of Mobile Phones scale, and the Smartphone Addiction Proneness Scale to French so that they are conceptually equivalent to their original versions. The cross-cultural adaptation process will be performed using the forward/backward method, a commonly used method [28-32] and recommended by the World Health Organization [33]. The translation will be considered valid when the tests performed by the participants are considered conclusive. This step is a prerequisite for the quantitative validation of the questionnaires.

## Methods

### Background

To assess the conceptual equivalence of the questionnaires, that is, whether the items in the original language have a similar meaning to the French version, we opted for the forward/backward translation method. This is the most commonly used technique for cross-cultural research, and we followed the recommendations of Epstein et al [34]. We have decided to validate these instruments for use exclusively in France.

### Ethics Approval

This study was approved by the Grenoble Alpes University Hospital Center and has received ethics approval from the South-East I Ethics Committee for the Protection of Individuals (approval 2022-A01943-40).

### Questionnaires

The 4 questionnaires targeted for the cross-cultural procedure were as follows. The Smartphone Addiction Scale [6] contains 33 items, and the response scale ranges from 1 (strongly disagree) to 6 (strongly agree). Each participant’s total score can range from 33 to 188 points, and a higher score indicates more problematic smartphone use. The Nomophobia Questionnaire [7] contains 20 items, and the response scale ranges from 1 (strongly disagree) to 7 (strongly agree); each participant’s total score can therefore vary from 20 to 140 points, and a higher score indicates more problematic smartphone use. The Problematic Use of Mobile Phones scale contains 20 items [8]. The response scale ranges from 1 (not at all in agreement) to 5 (totally in agreement), and each participant’s total score can vary from 20 to 100 points, and a higher score indicates more problematic smartphone use. Finally, the Smartphone Addiction Proneness Scale by Kim et al [9] contains 15 items. The response scale ranges from 1 (not at all in agreement) to 4 (totally in agreement), and each participant’s total score can vary from 15 to 60 points, and a higher score indicates more problematic smartphone use.

### Forward Translation

The first translation phase involved recruiting independent translators who were both 2 native French speakers bilingual in English and 2 native English speakers bilingual in French and asking them to translate the original English version into French. It was recommended that at least one of the translators knows the concept of the questionnaires to be measured and that at least one of them does not know the objective of the questionnaire. In this study, 4 qualified translators were involved: 2 translators were familiar with the concept of problematic smartphone use, while the other 2 were not. Two of the experts had backgrounds in health and psychology research: one was a novice and the other had prior experience in translation. At the end of the 4 translations, a single version was obtained after a reconciliation meeting of the 4 translators. Each questionnaire was checked for errors in spelling, grammar, punctuation, and translation of terminology and style against the original English version.

### Backward Translation

In the second phase, the French versions were retranslated into English by a native English speaker who had no clinical or medical expertise. The instructions that were given to the native English speaker were to translate each questionnaire literally. By comparing the translated English versions with the original questionnaires, the French translations were modified to be consistent with the originals. This step ensured the accuracy of the translations and highlighted any phrasing that could cause confusion.

### Linguistic Verification

In the third phase, 2 professionals familiar with the concept of problematic smartphone use were asked to comment and suggest modifications to the statements if necessary. We followed a universalist approach for equivalence, assessing conceptual, item, semantic, operational, measurement, and functional equivalence [35].

### Final Verification

The final verification phase involved performing a preliminary pilot test with participants involved in the problem being measured to assess the clarity, intelligibility, and acceptability of the translations. Between December 2022 and June 2023, 18 French native speaker participants—10 students older than 18 years (ie, 6 girls and 4 boys) and 8 adolescents aged 12-17 years (ie, 6 girls and 2 boys)—completed the questionnaires for the final version during face-to-face interviews. Each interview lasted a maximum of 30 minutes. For minor participants, consent to take part in the study was obtained from the parents, while adult participants gave their own consent. Participants could be in good health or experiencing any medical condition and they could come from rural or urban areas in France. During the interviews, a researcher asked the participants questions such as “Did you understand the instructions, the items, and the response scales?”; “How did you interpret this item?”; or “What would you suggest as a reformulation?” The principal investigator checked the proofs of the final version and corrected any errors.

### Data Analysis

To analyze the qualitative data from the translation process, we employed a thematic approach focused on assessing the conceptual equivalence of the translated items. Responses from the participants during the individual interviews were transcribed and systematically analyzed to identify elements that could pose comprehension issues. Particular attention was given to semantic discrepancies between the original and translated versions as well as suggestions for reformulation provided by the participants. The data were then coded according to the types of mistranslations identified (eg, item comprehension, appropriateness of terms used) and grouped into recurring themes. This approach enabled us to evaluate the clarity and acceptability of the translations as well as to adjust the problematic items.

## Results

### Characteristics of the Translators and the Participants

The age, gender, and specific cultural characteristics of the translators and the student and adolescent participants are detailed in Table 1.

**Table 1:** Age, gender, and cultural characteristics of the translators and the participants.

Translation type	Translators/participants (n)	Mean age (years)	Gender	Cultural characteristics
Forward translation	4 translators	25.5 (range 20-31)	2 females, 2 males	2 native English, 2 native French 2 were familiar with the concept of problematic smartphone use (eHealth and psychology) 2 were not familiar with the concept (economics and education) 2 were from the south of France and 2 were from the north
Backward translation	1 translator	28	1 female	Native English No clinical or medical expertise
Linguistic verification	2 experts	27.5 (range 24-31)	1 female, 1 male	2 native French
**Final verification**				
	8 adolescents	14.5 (range 12-17)	6 females, 2 males	Good health or experiencing any medical condition 5 from rural areas and 3 from urban areas in France
	10 students	19.8 (range 18-24)	6 females, 4 males	Good health or experiencing any medical condition 6 students in health sciences, 3 students in psychology, 1 student in philosophy

### Forward Translation

The 4 translators translated the titles, dimensions, instructions, response modalities, and statements of each questionnaire. These 4 translations were synthesized into a consensual version A. Concerning questionnaire dimensions, a few terms were more difficult to translate from English to French. For example, the dimension “withdrawal” of the Smartphone Addiction Scale, the Problematic Use of Mobile Phones scale, and the Smartphone Addiction Proneness Scale was translated as “sevrage.” For the Nomophobia Questionnaire, 1 dimension was more challenging to translate into French: “giving up convenience.” The term “convenience” can have several meanings in French, but its translation as “confort” (comfort) appears to be the most appropriate, especially when defining this dimension as the loss of the comfort provided by smartphone use. The concept of “craving” for the Nomophobia Questionnaire is particularly complex to define in French as it does not have a direct equivalent. In clinical terms, craving corresponds to a strong impulsive desire to use one’s object of addiction, which in our context is the respondent’s smartphone use. Therefore, in French, we have defined this dimension as “désir” (desire). For the other dimensions, the terms used in French were mostly similar.

For the items in the Smartphone Addiction Scale, rather than using the infinitive form of verbs as proposed in the original version, we translated them by systematically beginning the verb with the first-person singular conjugation. For example, for the original item “missing planned work due to smartphone use,” we translated it as “J’oublie du travail planifié à cause de l’utilisation du smartphone.” This will help participants better understand the statements. Three statements were more difficult to translate into French: “feeling pleasant or excited while using a smartphone” and “feeling impatient and fretful when I am not holding my smartphone.” The term “pleasant” was suggested in French as “calme” (calm), and since the term “impatient” does not have a direct equivalent in French, we propose the homograph “impatient.” The verb “to annoy” present in 2 items of the Nomophobia Questionnaire required more debate regarding its translation. This term can imply constraint, boredom, or irritation depending on the context; therefore, we chose to translate it as “contrariété” (contrariety) to preserve the statement and its dimension without distorting it. The statement “using a smartphone is more enjoyable than spending time with family or friends” from the Smartphone Addiction Proneness Scale also sparked discussions among translators because not everyone agreed on how to translate “enjoyable.” This term can signify a pleasant or amusing moment depending on the context. Here, we opted for the most common translation, “agréable” (pleasant). For the other items, the differences in translation were minor and did not alter the literal meaning of the statements.

### Backward Translation

Following this first translation, a native English translator backtranslated the questionnaires from the consensus version A into an English version B. This version B was compared to the original questionnaire in English. For the Smartphone Addiction Scale, 18 items were perfectly backtranslated, while 14 were slightly modified, but this did not alter their meaning and understanding. One item was retranslated, changing its meaning: “missing planned work due to smartphone use” was backtranslated as “I use my smartphone longer than planned.” We reworked this item in French to maintain its original meaning as “Je manque du travail planifié à cause de l’utilisation du smartphone.” For the Problematic Use of Mobile Phones scale, 17 statements were perfectly backtranslated, and the others underwent slight modifications without impacting their meaning and understanding. Backtranslation was even better for the Smartphone Addiction Proneness Scale, with 13 out of 15 items perfectly backtranslated and 2 having slight modifications without consequence. The backtranslation for the Nomophobia Questionnaire was satisfactory, with 10 out of 20 items varying slightly in wordings but without altering their meaning. The others were perfectly backtranslated. Thus, we obtained a translated version C for the 4 questionnaires.

### Linguistic Verification

The 2 researchers responsible for linguistic verification submitted 4 suggestions for conceptual modifications for the Smartphone Addiction Scale, 3 for the Nomophobia Questionnaire, and 1 for the Problematic Use of Mobile Phones scale, some of which were incorporated into the final C version. No additional modifications were suggested for the Smartphone Addiction Proneness Scale.

### Final Verification

In this final step, feedback from adolescents and students was minimal; therefore, we produced a final version D based on their input. Overall, they understood all the instructions, items, and response modalities well. Participants encountered no comprehension problems except for 3 items. For the Nomophobia Questionnaire, 2 students reported a comprehension difficulty with item 18. In English, it refers to checking “online connections and networks,” and we translated it as “online notifications and networks.” However, “online networks” made less sense than “social networks” for these students. We therefore modified the item. In addition, item 6 of the Problematic Use of Mobile Phones scale was difficult for one student to understand, as the translators had translated it by mixing the present and past tenses. We therefore modified the item from “J’ai pensé par le passé qu’il n’était pas normal de passer autant de temps sur le smartphone comme je le fais” to “Il m’est arrivé de penser qu’il n’était pas normal de passer autant de temps que moi à utiliser un smartphone.” In this way, students gained a better understanding of the item. Finally, item 22 of the Smartphone Addiction Scale had to be clarified, as 2 participants did not understand what “losing a friend” meant. In fact, the meaning of loss is important in French, as it could mean literally losing a friend through death or signify a friendship breakup. We have therefore clarified at the end of the item that this refers to friendship breakup in this context. The final translated versions are listed in Tables 2-5.

**Table 2:** Problematic Use of Mobile Phones scale translation.

Item number	Item description	Response methods				
		1^a^	2	3	4	5^b^
1	ET^c^: When I decrease the amount of time spent using my cell phone I feel less satisfied. FT^d^: Quand je réduis le temps passé sur mon smartphone, je me sens moins satisfait·e.					
2	ET: I need more time using my cell phone to feel satisfied than I used to need. FT: J’ai besoin de plus de temps d’utilisation de mon smartphone afin de me sentir aussi satisfait·e qu’auparavant.					
3	ET: When I stop using my cell phone, I get moody and irritable. FT: Quand j’arrête d’utiliser mon smartphone, je deviens de mauvaise humeur et irritable.					
4	ET: It would be very difficult emotionally to give up my cell phone. FT: Ce serait difficile émotionnellement de renoncer à mon smartphone.					
5	ET: The amount of time I spend using my cell phone keeps me from doing other important work. FT: Le temps passé sur mon smartphone m’empêche de faire d’autres tâches importantes.					
6	ET: I have thought in the past that it is not normal to spend as much time using a cell phone as I do. FT: Il m’est arrivé·e de penser qu’il n’était pas normal de passer autant de temps que moi à utiliser un smartphone.					
7	ET: I think I might be spending too much time using my cell phone. FT: Je pense que je passe trop de temps sur mon smartphone.					
8	ET: People tell me I spend too much time using my cell phone. FT: Les gens me disent que je passe trop de temps sur mon smartphone.					
9	ET: When I am not using my cell phone, I am thinking about using it or planning the next time I can use it. FT: Quand je n’utilise pas mon smartphone, je pense à l’utiliser ou à prévoir ma prochaine utilisation.					
10	ET: I feel anxious if I have not received a call or message in some time. FT: Je me sens anxieux·se si je n’ai pas reçu d’appel ou de message depuis un certain moment.					
11	ET: I have ignored the people I’m with in order to use my cell phone. FT: J’ai ignoré les personnes avec qui j’étais pour utiliser mon smartphone.					
12	ET: I have used my cell phone when I knew I should be doing work/schoolwork. FT: J’ai utilisé mon téléphone alors que je savais que je devais travailler/faire des devoirs.					
13	ET: I have used my cell phone when I knew I should be sleeping. FT: J’ai déjà utilisé mon smartphone alors que je savais que je devais dormir.					
14	ET: When I stop using my cell phone because it is interfering with my life, I usually return to it. FT: Quand j’arrête d’utiliser mon smartphone parce qu’il interfère avec ma vie, je finis généralement par l’utiliser à nouveau.					
15	ET: I have gotten into trouble at work or school because of my cell phone use. FT: J’ai eu des problèmes au travail ou à l’école à cause de l’utilisation de mon smartphone.					
16	ET: At times, I find myself using my cell phone instead of spending time with people who are important to me and want to spend time with me. FT: Parfois, je me retrouve à utiliser mon smartphone au lieu de passer du temps avec des personnes qui sont importantes pour moi et qui souhaitent passer du temps avec moi.					
17	ET: I have used my cell phone when I knew it was dangerous to do so. FT: J’ai utilisé mon téléphone alors que je savais qu’il était dangereux de le faire.					
18	ET: I have almost caused an accident because of my cell phone use. FT: J’ai presque causé un accident à cause de l’utilisation de mon smartphone.					
19	ET: My cell phone use has caused me problems in a relationship. FT: L’utilisation de mon smartphone a causé des problèmes dans une relation.					
20	ET: I have continued to use my cell phone even when someone asked me to stop. FT: J’ai continué d’utiliser mon smartphone, même quand quelqu’un m’a demandé d’arrêter.					

^a^English translation: strongly disagree; French translation: fortement en désaccord.

^b^English translation: strongly agree; French translation: fortement en accord.

^c^ET: English translation.

^d^FT: French translation.

**Table 3:** Smartphone Addiction Scale translation.

Item number	Item description	Response methods					
		1^a^	2^b^	3^c^	4^d^	5^e^	6^f^
1	ET^g^: Missing planned work due to smartphone use. FT^h^: Je manque du travail planifié à cause de l’utilisation du smartphone.						
2	ET: Having a hard time concentrating in class while doing assignments or while working due to smartphone use. FT: J’ai des difficultés à me concentrer en classe, pendant mes devoirs ou pendant le travail à cause de l’utilisation du smartphone.						
3	ET: Experiencing lightheadedness or blurred vision due to excessive smartphone use. FT: Je ressens des vertiges ou une vision floue à cause de l’utilisation excessive du smartphone.						
4	ET: Feeling pain in the wrist or at the back of the neck while using a smartphone. FT: Je ressens de la douleur dans les poignets ou derrière le cou pendant que j’utilise un smartphone.						
5	ET: Feeling tired and lacking adequate sleep due to excessive smartphone use. FT: Je me sens fatigué et en manque de sommeil suffisant à cause de l’utilisation excessive du smartphone.						
6	ET: Feeling calm or cozy while using a smartphone. FT: Je me sens calme et réconforté·e quand j’utilise un smartphone.						
7	ET: Feeling pleasant or excited while using a smartphone. FT: Je me sens calme ou excité·e en utilisant le smartphone.						
8	ET: Feeling confident while using a smartphone. FT: Je me sens confiant·e en utilisant le smartphone.						
9	ET: Being able to get rid of stress with a smartphone. FT: Je suis capable de me débarrasser du stress avec un smartphone.						
10	ET: There is nothing more fun to do than using my smartphone. FT: Il n’y a rien de plus amusant que d’utiliser mon smartphone.						
11	ET: My life would be empty without my smartphone. FT: Ma vie serait vide sans mon smartphone.						
12	ET: Feeling most liberal while using a smartphone. FT: Je me sens libre quand j’utilise un smartphone.						
13	ET: Using a smartphone is the most fun thing to do. FT: Utiliser un smartphone est la chose la plus amusante à faire.						
14	ET: Won’t be able to stand not having a smartphone. FT: Je ne supporterais pas de ne pas avoir de smartphone.						
15	ET: Feeling impatient and fretful when I am not holding my smartphone. FT: Je me sens impatient·e et irrité·e quand je ne tiens pas mon smartphone.						
16	ET: Having my smartphone in my mind even when I am not using it. FT: Je pense à mon smartphone même lorsque je ne l’utilise pas.						
17	ET: I will never give up using my smartphone even when my daily life is already greatly affected by it. FT: Je ne renoncerais jamais à utiliser mon smartphone, même si ma vie quotidienne en est déjà fortement affectée.						
18	ET: Getting irritated when bothered while using my smartphone. FT: Je me sens irrité·e quand on me dérange pendant que j’utilise mon smartphone.						
19	ET: Bringing my smartphone to the toilet even when I am in a hurry to get there. FT: J’apporte mon smartphone aux toilettes même lorsque je suis pressé·e d’y aller.						
20	ET: Feeling great meeting more people via smartphone use. FT: Je me sens bien en rencontrant plus de personnes par le biais de l’utilisation de mon smartphone.						
21	ET: Feeling that my relationships with my smartphone buddies are more intimate than my relationships with my real-life friends. FT: J’ai l’impression que mes relations virtuelles sont plus intimes que mes relations avec mes amis de la vie réelle.						
22	ET: Not being able to use my smartphone would be as painful as losing a friend. FT: Ne pas pouvoir utiliser mon smartphone serait aussi douloureux que de perdre un ami (rupture amicale).						
23	ET: Feeling that my smartphone buddies understand me better than my real-life friends. FT: J’ai l’impression que mes amis virtuels me comprennent mieux que mes amis de la vie réelle.						
24	ET: Constantly checking my smartphone so as not to miss conversations between other people on Twitter or Facebook. FT: Je vérifie constamment mon smartphone afin de ne manquer aucune conversation entre d’autres personnes sur Twitter ou Facebook.						
25	ET: Checking social networking service sites like Twitter or Facebook right after waking up. FT: Je vérifie les réseaux sociaux comme Twitter ou Facebook juste après le réveil.						
26	ET: Preferring talking with my smartphone buddies to hanging out with my real-life friends or with the other members of my family. FT: Je préfère discuter avec mes amis virtuels plutôt que passer du temps avec mes amis de la vie réelle ou avec les membres de ma famille.						
27	ET: Preferring searching from my smartphone to asking other people. FT: Je préfère faire mes recherches à partir de mon smartphone plutôt que de demander à d’autres personnes.						
28	ET: My fully charged battery does not last for one whole day. FT: Ma batterie pleinement chargée ne dure pas une journée entière.						
29	ET: Using my smartphone longer than I had intended. FT: J’utilise mon smartphone plus longtemps que prévu.						
30	ET: Feeling the urge to use my smartphone again right after I stopped using it. FT: Je ressens le besoin d’utiliser à nouveau mon smartphone juste après avoir arrêté de l’utiliser.						
31	ET: Having tried time and again to shorten my smartphone use time but failing all the time. FT: J’ai essayé à plusieurs reprises de réduire le temps d’utilisation de mon smartphone mais j’échoue à chaque fois.						
32	ET: Always thinking that I should shorten my smartphone use time. FT: Je pense toujours que je devrais réduire mon utilisation du smartphone.						
33	ET: The people around me tell me that I use my smartphone too much. FT: Les gens autour de moi me disent que j’utilise trop mon smartphone.						

^a^English translation: strongly disagree; French translation: fortement en désaccord.

^b^English translation: disagree; French translation: en désaccord.

^c^English translation: weakly disagree; French translation: un peu en désaccord.

^d^English translation: weakly agree; French translation: un peu en accord.

^e^English translation: agree; French translation: en accord.

^f^English translation: strongly agree; French translation: fortement en accord.

^g^ET: English translation.

^h^FT: French translation.

**Table 4:** Nomophobia Questionnaire translation.

Item number		Item description	Response methods						
			1^a^	2	3	4	5	6	7^b^
1		ET^c^: I would feel uncomfortable without constant access to information through my smartphone. FT^d^: Je me sentirais mal à l’aise sans l’accès constant à l’information au travers de mon smartphone.							
2		ET: I would be annoyed if I could not look information up on my smartphone when I wanted to do so. FT: Je me sentirais contrarié·e si je ne pouvais pas chercher de l’information sur mon smartphone quand je le souhaite.							
3		ET: Being unable to get the news (eg, happenings, weather, etc) on my smartphone would make me nervous. FT: Être incapable d’avoir accès aux nouvelles (eg, actualités, météo, etc.) sur mon smartphone me rendrait nerveux·se.							
4		ET: I would be annoyed if I could not use my smartphone and/or its capabilities when I wanted to do so. FT: Je serais contrarié·e si je ne pouvais pas utiliser mon smartphone et/ou ses fonctionnalités quand je le souhaite.							
5		ET: Running out of battery in my smartphone would scare me. FT: Ne plus avoir de batterie sur mon smartphone me ferait peur.							
6		ET: If I were to run out of credits or hit my monthly data limit, I would panic. FT: Si je me retrouvais sans crédits ou si j’atteignais ma limite de données mensuelle, je paniquerais.							
7		ET: If I did not have a data signal or could not connect to Wi-Fi, then I would constantly check to see if I had a signal or could find a Wi-Fi network. FT: Si je n’avais pas de signal réseau ou ne pouvais pas me connecter au Wi-Fi, je vérifierais constamment si j’ai du réseau ou si je peux trouver un réseau Wi-Fi.							
8		ET: If I could not use my smartphone, I would be afraid of getting stranded somewhere. FT: Si je ne pouvais pas utiliser mon smartphone, j’aurais peur de rester bloqué·e quelque part.							
9		ET: If I could not check my smartphone for a while, I would feel a desire to check it. FT: Si je ne pouvais pas consulter mon smartphone pendant un certain temps, j’aurais envie de le consulter.							
**ET:** **If I did not have my smartphone with me, FT:** **Si je n’avais pas mon smartphone avec moi,**									
	10	ET: I would feel anxious because I could not instantly communicate with my family and/or friends. FT: Je me sentirais anxieux·se parce que je ne pourrais pas communiquer instantanément avec ma famille et/ou mes amis.							
	11	ET: I would be worried because my family and/or friends could not reach me. FT: Je serais inquiet·e parce que ma famille et/ou mes amis ne pourraient pas me joindre.							
	12	ET: I would feel nervous because I would not be able to receive text messages and calls. FT: Je me sentirais nerveux·se de ne pas pouvoir recevoir des messages ou des appels.							
	13	ET: I would be anxious because I could not keep in touch with my family and/or friends. FT: Je me sentirais anxieux·se parce que je ne pourrais pas rester en contact avec ma famille et/ou mes amis.							
	14	ET: I would be nervous because I could not know if someone had tried to get a hold of me. FT: Je serais nerveux·se parce que je ne pourrais pas savoir si quelqu’un a essayé de me contacter.							
	15	ET: I would feel anxious because my constant connection to my family and friends would be broken. FT: Je me sentirais anxieux·se parce que ma connexion constante avec ma famille et mes amis serait rompue.							
	16	ET: I would be nervous because I would be disconnected from my online identity. FT: Je serais nerveux·se parce que je serais déconnecté·e de mon identité en ligne.							
	17	ET: I would be uncomfortable because I could not stay up-to-date with social media and online networks. FT: Je serais mal à l’aise parce que je ne pourrais pas être à jour avec les réseaux sociaux ou les réseaux en ligne.							
	18	ET: I would feel awkward because I could not check my notifications for updates from my connections and online networks. FT: Je me sentirais mal à l’aise parce que je ne pourrais pas vérifier mes notifications et réseaux sociaux.							
	19	ET: I would feel anxious because I could not check my email messages. FT: Je me sentirais anxieux·se parce que je ne pourrais pas vérifier mes emails.							
	20	ET: I would feel weird because I would not know what to do. FT: Je me sentirais mal à l’aise car je ne saurais pas quoi faire.							

^a^English translation: strongly disagree; French translation: fortement en désaccord.

^b^English translation: strongly agree; French translation: fortement en accord.

^c^ET: English translation.

^d^FT: French translation.

**Table 5:** Smartphone Addiction Proneness Scale translation.

Item number	Item description	Response methods			
		1^a^	2^b^	3^c^	4^d^
					
1	ET^e^: My school grades dropped due to excessive smartphone use. FT^f^: Mes notes scolaires ont baissé à cause d’une utilisation excessive du smartphone.				
2	ET: I have a hard time doing what I have planned (study, do homework, or go to afterschool classes) due to using smartphone. FT: J’ai du mal à faire ce que j’avais prévu (étudier, faire mes devoirs, ou aller aux activités scolaires) à cause de l’utilisation du smartphone.				
3	ET: People frequently comment on my excessive smartphone use. FT: Les gens commentent fréquemment mon utilisation excessive du smartphone.				
4	ET: Family or friends complain that I use my smartphone too much. FT: Ma famille ou mes amis se plaignent que j’utilise trop mon smartphone.				
5	ET: My smartphone does not distract me from my studies (reversed item). FT: Mon smartphone ne me distrait pas de mes études. (énoncé inversé)				
6	ET: Using a smartphone is more enjoyable than spending time with family or friends. FT: Utiliser un smartphone est plus agréable que de passer du temps avec ma famille ou mes amis.				
7	ET: When I cannot use a smartphone, I feel like I have lost the entire world. FT: Quand je ne peux pas utiliser un smartphone, j’ai l’impression d’avoir perdu le monde entier.				
8	ET: It would be painful if I am not allowed to use a smartphone. FT: Ce serait douloureux si je n’étais pas autorisé·e à utiliser un smartphone.				
9	ET: I get restless and nervous when I am without a smartphone. FT: Je deviens agité·e et nerveux·se quand je suis sans smartphone.				
10	ET: I am not anxious even when I am without a smartphone (reversed item). FT: Je ne suis pas anxieux·se, même quand je suis sans smartphone. (énoncé inversé)				
11	ET: I panic when I cannot use my smartphone. FT: Je panique quand je ne peux pas utiliser mon smartphone.				
12	ET: I try cutting my smartphone usage time, but I fail. FT: J’essaie de réduire le temps d’utilisation de mon smartphone, mais j’échoue.				
13	ET: I can control my smartphone usage time (reversed item). FT: Je peux contrôler le temps d’utilisation de mon smartphone. (énoncé inversé)				
14	ET: Even when I think I should stop, I continue to use my smartphone too much. FT: Même lorsque je pense que je devrais arrêter, je continue de trop utiliser mon smartphone.				
15	ET: Spending a lot of time on my smartphone has become a habit. FT: Passer beaucoup de temps sur mon smartphone est devenu une habitude.				

^a^English translation: strongly disagree; French translation: fortement en désaccord.

^b^English translation: disagree; French translation: en désaccord.

^c^English translation: agree; French translation: en accord.

^d^English translation: strongly agree; French translation: fortement en accord.

^e^ET: English translation.

^f^FT: French translation.

## Discussion

### Principal Findings

The Problematic Use of Mobile Phones scale, the Smartphone Addiction Scale, the Smartphone Addiction Proneness Scale, and the Nomophobia Questionnaire for young adult students and adolescents have been developed and validated in English to measure problematic smartphone use, but they are not available in French. In this study, we provide their translations and linguistic validation in French.

Translating questionnaires into other languages can be complex, as terms in one language can be interpreted in different ways in another, leading to errors in meaning. For this reason, we have followed the rigorous and standardized steps of linguistic validation defined by the forward/backward method [34].

The results of the translations showed that after 4 stages of translation, the content of the French questionnaires was identical to that of the English ones. The translators, adolescents, and young adults from different educational/professional and geographical backgrounds did not reveal any differences in understanding, suggesting that the cultural adaptation of the questionnaires was respected. These translations suggest that cross-cultural adaptation can be used for comparative purposes in other languages such as English, Portuguese, Arabic, or German.

However, 3 limitations need to be addressed in this study. First, the number of adolescents and young adult participants was less than 20. Although it would be more robust to interview more participants, the minimum number of participants for linguistic validation is not clearly defined [35]. Moreover, the adolescents and young adults provided only few comments on the clarity and understanding of the questionnaires. Second, although 4 volunteer translators were consulted, a more diversified panel of experts would have enabled us to avoid any cultural bias in the translation of the questionnaires. We were able to bring together 4 translators of different genders (ie, 2 men and 2 women) and from different disciplines (ie, eHealth, psychology, education, and economics) who did not have professional knowledge of the concept of problematic smartphone use. Despite these considerations, France is a country with a rich cultural diversity, which may include some regional and linguistic differences. We could have included native experts from different regions with marked linguistic peculiarities (eg, Brittany, Corsica). It is important to note that the 4 translators selected came from different regions of France, which ensures that we have reduced at least some of the cultural biases in translation. The last limitation was that this study focuses solely on linguistic validation without demonstrating quantitative validation. According to Terwee and colleagues [36], the translation process is the first necessary step for validation, but it is essential to verify content validity, internal consistency, criterion validity, construct validity, and reproducibility. This is particularly important here because problematic smartphone use, as measured by the questionnaires, is often assessed across multiple dimensions such as tolerance, withdrawal symptoms, overuse, or physical and psychological consequences [3]. These validation steps, especially regarding psychometric properties, are essential and must be completed before any use of these questionnaires in French. They can then be reliably used to better understand problematic smartphone use among French speakers and for conducting cross-cultural comparisons. These questionnaires will be relevant for gaining a better understanding of how problematic smartphone use is characterized among French adults and adolescents and can be utilized for screening and potentially monitoring patients with conditions associated with problematic smartphone use [5,37].

### Conclusion

We succeeded in adapting and effectively translating 4 questionnaires assessing problematic smartphone use in a French-speaking context. This step remains a prerequisite to validating the questionnaires, which can serve as valuable research instruments for investigating and addressing issues related to problematic smartphone use in French-speaking countries and for making international comparisons. Given that problematic smartphone use is an emerging concern with significant health impacts [1], it is imperative that researchers and health care professionals use reliable measurement instruments.

## Abbreviations

DSM: Diagnostic and Statistical Manual of Mental Disorders
